# Hemodialysis catheter-associated superior vena cava syndrome and pulmonary embolism: a case report and review of the literature

**DOI:** 10.1186/s13104-016-2043-1

**Published:** 2016-04-23

**Authors:** Sritika Thapa, Peter B. Terry, Biren B. Kamdar

**Affiliations:** Division of Hospital Medicine, Johns Hopkins Bayview Medical Center, Johns Hopkins University School of Medicine, 5200 Eastern Ave., MFL Bldg, West Tower 6th Floor CIMS Suite, Baltimore, MD 21224 USA; Division of Pulmonary and Critical Care Medicine, Johns Hopkins Hospital, Johns Hopkins University School of Medicine, Baltimore, MD USA; Division of Pulmonary and Critical Care Medicine, David Geffen School of Medicine at UCLA, Los Angeles, CA USA

**Keywords:** Hemodialysis, Catheter, Thrombosis, Superior vena cava syndrome, Pulmonary embolism

## Abstract

**Background:**

Hemodialysis (HD) catheters are frequently inserted into the superior vena cava (SVC), and can lead to thrombotic complications. However, to our knowledge, HD catheter-related thrombosis leading to subsequent SVC syndrome, bacteremia, and pulmonary emboli has not been described.

**Case presentation:**

A 28-year-old dialysis-dependent woman with IgA nephropathy developed facial swelling, head pressure, headache, nausea, dizziness and fever 6 weeks after right internal jugular (IJ) HD catheter placement. Chest and neck imaging demonstrated a non-occlusive thrombus surrounding the HD catheter and extending from the SVC to the junction of the right IJ and right subclavian veins, confirming thrombosis-associated SVC syndrome. Intravenous (IV) anticoagulation was initiated, as well as IV vancomycin for *Staphylococcus epidermidis* bacteremia. Despite prompt intravenous anticoagulation, 9 and 12 days after initial presentation she developed catheter-associated pulmonary embolism (PE) and PE-associated pulmonary infarction, respectively. Hypercoagulable workup was negative. The HD catheter was eventually replaced, HD resumed, and the patient was transitioned from intravenous to oral anticoagulation and discharged. Nine months later, she underwent successful renal transplantation.

**Conclusion:**

SVC syndrome and pulmonary embolism are potential consequences of HD catheter-related thrombosis. Given the frequency of HD catheter placement, physicians should be aware of these potential complications in any patient with HD catheter-related thrombosis.

## Background

In 2012, 61 % of patients with incident end-stage renal disease (ESRD) were initiated on hemodialysis (HD) via catheter alone (i.e., without a fistula or arteriovenous graft) [1]. When inserted into the lumen of the superior vena cava (SVC), these HD catheters have been shown to be associated with catheter-related thrombosis, SVC syndrome, bacteremia and thromboembolism [2–11]. However, to our knowledge, the co-occurrence of these complications in the setting of HD catheter placement has not been reported.

## Case presentation

A 28-year-old Asian female hairstylist with hypertension and ESRD secondary to IgA nephropathy presented to an outside hospital with one week of facial swelling, head pressure that worsened when lying on the right side, headache when bending forward, nausea, dizziness and fever (Table 1). Six weeks prior to presentation, she initiated hemodialysis via a tunneled right internal jugular (IJ) HD catheter. Her substance use, social, and family history were unremarkable. Contrast-enhanced computed tomography (CT) of the chest and neck performed on the day of admission demonstrated the HD catheter tip in the inferior right atrium, with thrombus formed around the HD catheter and extending along the catheter from the junction of the right IJ and right subclavian vein, through the SVC and to the level of the cavoatrial junction. In the setting of SVC thrombus, she was initiated on a continuous intravenous (IV) heparin infusion.

**Table 1:** Timeline of clinical events

Time	Event
Day 0	28yo woman with end-stage renal disease in the setting of IgA nephropathy undergoes right IJ tunneled HD catheter placed for initiation of hemodialysis
Day 47	Presents to outside hospital with SVC syndrome and started on intravenous heparin
Day 51	Transferred to our hospital with SVC syndrome and bacteremia (on IV heparin and IV vancomycin) for consideration of advanced interventions involving the HD catheter
Day 55	Due to the persistence of thrombus-associated SVC syndrome, HD catheter is removed and replaced; IV vancomycin and IV heparin are continued, and coumadin is initiated
Day 60	Develops right-sided chest pain with fever; CT angiography demonstrates R-sided pulmonary embolism
Day 63	Develops pleuritic chest pain with blood tinged sputum; CT chest demonstrates pulmonary infarction
Day 69	Resolution of symptoms; discharged with six-month course of coumadin, 28-day course of vancomycin, and outpatient hemodialysis
Day 268 (approximately 9 months after discharge)	Underwent renal transplant

Based on blood cultures obtained on admission to the outside hospital (4 days prior to transfer to our hospital), the patient was found to have *Staphylococcus epidermidis* bacteremia, prompting initiation of IV vancomycin. Repeat blood cultures were negative. Since the thrombus was situated proximal to the catheter lumen, hemodialysis was considered safe and continued without incident. Given the need for possible advanced interventions for the tunneled HD catheter, 4 days after admission she was transferred to our tertiary care hospital for further management.

Upon transfer, her vital signs were: temperature 38.3 ℃, heart rate 96 beats per minute, blood pressure of 134/61 mmHg, respiratory rate of 18 breaths per minute and oxygen saturation of 99 % on room air. Her physical exam was notable for diffuse facial swelling and neck fullness. The heparin drip, vancomycin, analgesics and hemodialysis were continued. Warfarin was considered but held in anticipation of possible HD catheter replacement. A hypercoagulable workup was negative.

**Fig. 1:**
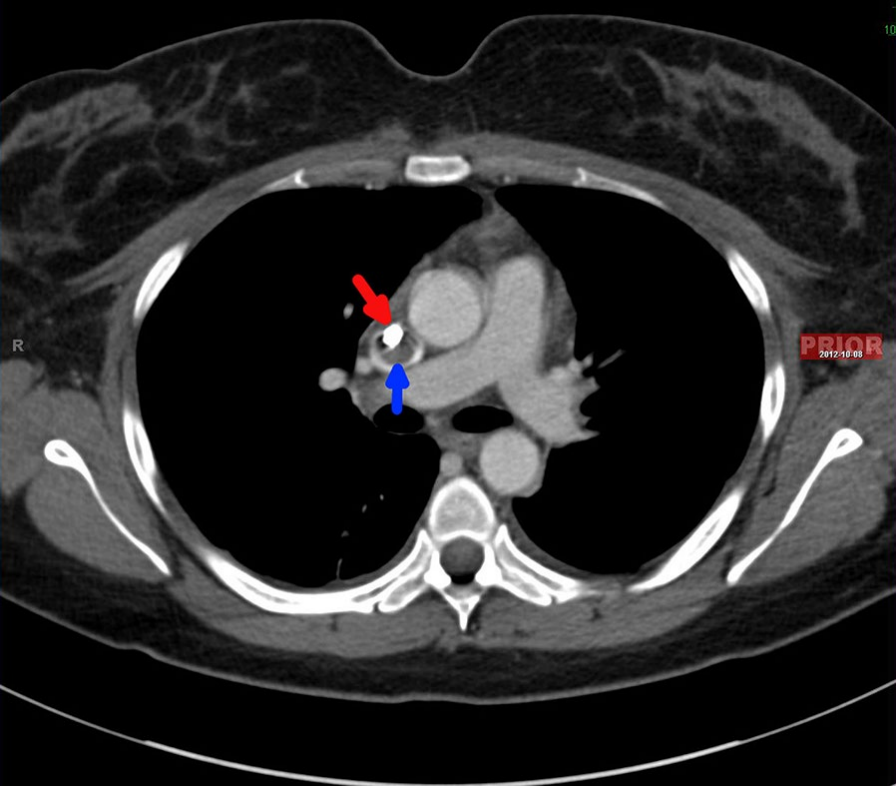
CT chest with contrast (*transverse view*) demonstrating an HD catheter (*bright white* shown by *red arrow*) with surrounding thrombus in the superior vena cava (*blue arrow*)

**Fig. 2:**
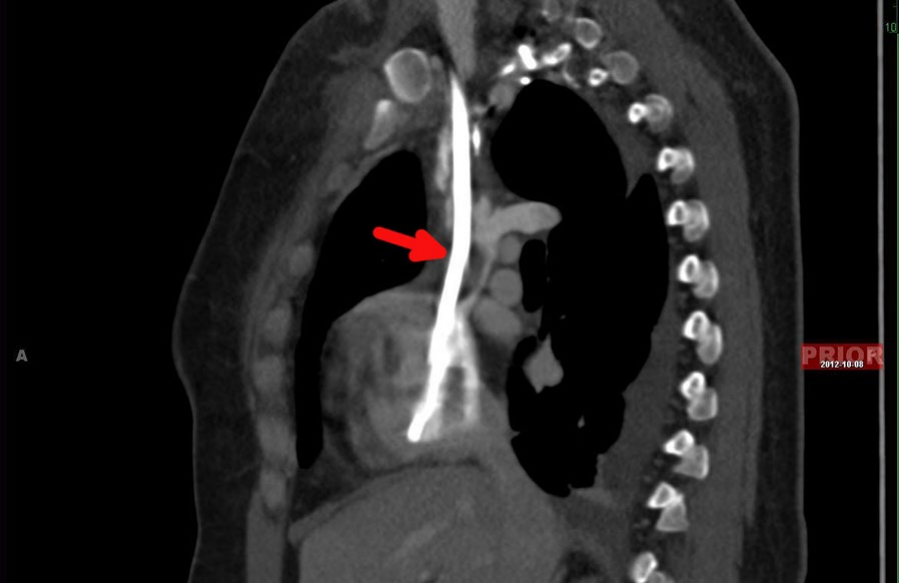
CT chest with contrast (*sagittal view*) demonstrating catheter in the SVC (*bright white* shown by *red arrow*) with surrounding thrombus

In the first 4 days at our hospital, the symptoms of SVC syndrome persisted despite continuous IV heparin, with worsening headache and facial pressure along with persistent fever despite negative blood cultures. Repeat contrasted CT of the chest demonstrated unchanged adherent, non-occlusive thrombus around the catheter without extension beyond the SVC (Figs. 1, 2). Pharmacological thrombolysis was considered but not performed in the setting of bacteremia, out of concern for bacterial shedding of a possibly infected clot (despite negative repeat blood cultures). Given the persistent thrombus-associated SVC syndrome with worsening headache and possibility of an infected thrombus with persistent fever, the interventional radiology team removed and replaced the right IJ HD catheter without incident, using ultrasound- and fluoroscopy-guided methods. Using digital subtraction angiography (DSA) during fluoroscopy, an eccentric, adherent intraluminal thrombus measuring approximately 2 cm was visualized on the right lateral aspect of the SVC. After replacement of the HD catheter, both the IV heparin drip and vancomycin along with analgesia were resumed, and warfarin was initiated.

**Fig. 3:**
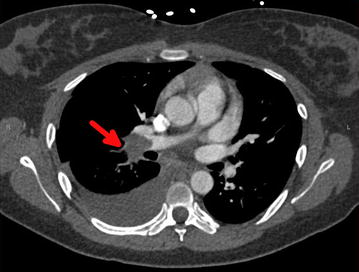
Computed tomography angiography (CTA) of the chest (*transverse view*) demonstrating thrombus in the *right* pulmonary artery (*red arrow*)

Nine days into admission and 5 days after new HD catheter replacement, the patient developed acute right-sided pleuritic chest pain with high-grade fevers. Computed tomography angiography (CTA) of the chest demonstrated an interval decrease in size of the SVC thrombus, along with new pulmonary embolism involving the right main, middle, and lower lobe pulmonary arteries (Fig. 3). Because the patient was hemodynamically stable and had no signs of right ventricular dysfunction on transthoracic echocardiogram, thrombolysis was not considered. Regarding her fever, repeat blood cultures were negative, lending suspicion to fever secondary to clot burden. However, out of concern for infected thrombus in a patient undergoing kidney transplant evaluation, vancomycin was continued based on a recommendation of the infectious disease team. The patient was therefore continued on continuous IV heparin, warfarin, IV vancomycin, analgesics, and antipyretics.

**Fig. 4:**
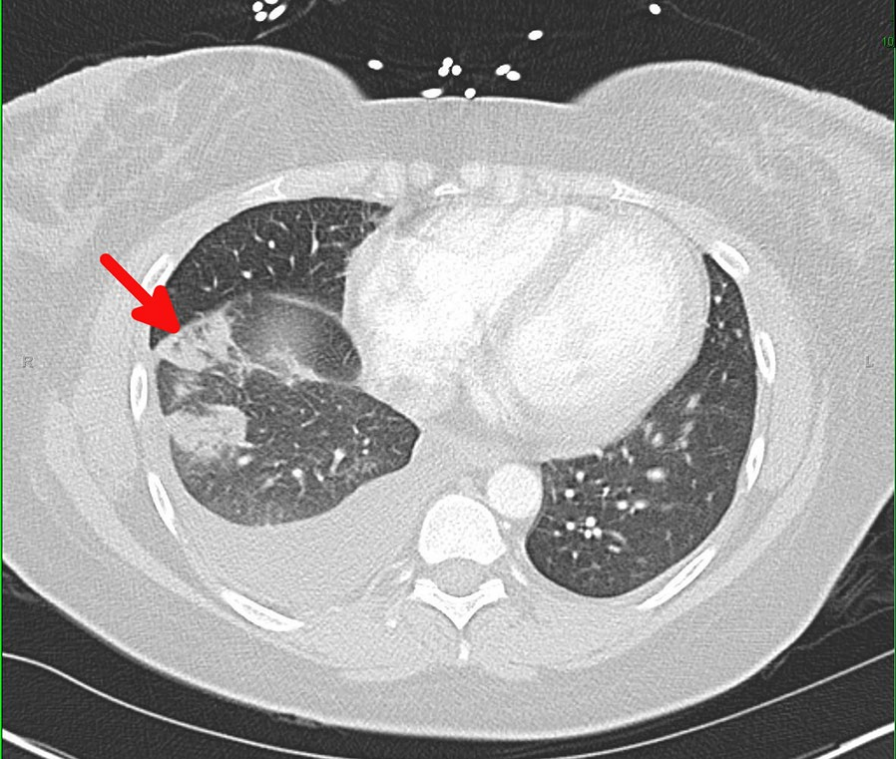
CT chest without contrast (*transverse view*) demonstrating a *right* lower lobe wedge-shaped pulmonary infarction (*red arrow*)

Three days after the pulmonary embolism, the patient again developed pleuritic chest pain, but this time accompanied by blood-tinged sputum. A non-contrasted CT scan of the chest showed multifocal peripheral wedge-shaped ground glass areas in the right lower and middle lobes, consistent with pulmonary infarction (Fig. 4). In the setting of a subtherapeutic international normalized ratio (INR) for prothrombin time (PT), both heparin and warfarin were continued. Once the INR reached therapeutic levels (goal range 2.0–3.0), the heparin infusion was discontinued.

Eighteen days after transfer to our hospital and 22 days after her initial hospital admission, the patient’s symptoms had remitted completely and she was discharged with prescriptions for a 6-month course of coumadin, completion of a 28-day course of vancomycin (starting from the day of her catheter removal), and oral pain medications. Outpatient hemodialysis continued uneventfully through the new-tunneled HD catheter. Her INR was monitored closely with no adverse events noted. Nine months after discharge, she underwent successful renal transplantation.

## Discussion

This case involved a young female patient who experienced three particularly extreme complications of HD catheter-associated thrombosis: superior vena cava (SVC) syndrome, bacteremia and pulmonary embolism (PE). HD catheters, which are often inserted into the internal jugular vein, are predisposed to catheter-related thrombosis. As compared to other central venous catheters (CVCs), it is believed that HD catheters may be associated with an increased risk of thrombosis-related complications, due to being used for hemodialysis, having longer and thicker lumens, longer placement duration, and/or differences in blood flow, however this relationship is unknown. Similar to CVCs, risk factors for HD catheter-related thrombosis include catheter-related characteristics including catheter caliber-to-vein ratio (i.e., 45 % or less associated with decreased VTE risk [12]), venipuncture-associated trauma (which can be reduced with ultrasound guidance [13]), and catheter position (i.e., higher risk if catheter tip is in the brachiocephalic vein or proximal SVC versus distal to SVC [14]), and patient-related characteristics including vein caliber, malignancy, prior thromboembolism, and a hypercoagulable state [15]. The mechanism underlying HD catheter-associated thrombosis is poorly understood, however factors such as recurrent vascular access, platelet dysfunction, endothelial factors, inflammation, and clotting abnormalities have been suggested [4].

While we could not be certain that our patient had a predisposing prothrombotic state, her history of IgA nephropathy and isolated proteinuria may have been associated with the thromboembolic event, given case reports suggesting an association of combined IgA nephropathy, malignancy, nephrotic syndrome, and heavy proteinuria with renal vein thrombosis, thrombotic microangiopathy, left ventricular thrombus, and PE [16–19]. However, patients with isolated IgA nephropathy, as compared to those with membranous nephropathy (MN) or focal segmental glomerulosclerosis (FSGS), have been shown to have a relatively low risk of thromboembolism [16, 20]. Hence, given that our patient had proteinuria without nephrotic syndrome in the setting of IgA nephropathy, we were less sure that IgA nephropathy played a substantial role, if any, in her development of HD catheter-related thrombosis.

Regarding the PE presented in this case, prior studies of thrombosed venous catheters have found pulmonary emboli pathologically similar to the thrombosis in the SVC [21, 22]. Additionally, thrombus dislodgement has been reported immediately following catheter removal using transesophageal echocardiographic imaging [23]. In this specific case, we theorized the patient’s PE occurred secondary to slow migration of thrombus off the catheter or SVC vessel wall, or to its dislodgement during HD catheter removal, and not due to non-catheter-related thrombus formation. Given that our patient had a PE 5 days after HD catheter removal, it seems more likely that slow thrombus migration explained her PE more so than catheter removal-related thrombus dislodgement. Nevertheless, we cannot be certain of the precise mechanism.

At this time, there are no published strategies regarding prevention of venous thrombosis and thromboembolism in patients with HD catheters. Large randomized controlled trials (RCTs) involving prophylactic anticoagulation for cancer patients with CVCs have been published, including a recent meta-analysis of nine RCTs demonstrating unclear benefits and risks of thromboprophylaxis in this patient population. To our knowledge, trials in patients without cancer, such as the patient presented in this case, have not been performed [15, 24]. Until more data surface in this area, practitioners can continue to perform standard catheter care, including regular flushing with heparin or saline to maintain patency, and consideration of silicon- or polyurethane-based catheters instead of those made from polyethylene. However, these strategies may not necessarily be associated with lower thrombosis risk [25].

As per guidelines by the American College of Chest Physicians (ACCP), in the setting of catheter-associated upper extremity DVTs, catheters no longer required can be removed. Available data do not suggest use of anticoagulation prior to catheter removal, but do recommend anticoagulation for 3 months following removal. However, when catheters are functional and required, they can remain in place, and anticoagulation should be continued for the duration of catheter placement [26]. Since we were not aware of the presence of an upper-extremity DVT in the above case, these guidelines do not specifically apply to our patient. Moreover, specific recommendations regarding catheter-related SVC or right atrial thrombosis with or without PE have not been published. In case reports and small studies involving patients similar to ours, anticoagulant therapy with low molecular weight heparin (in patients without ESRD) or unfractionated heparin has been administered, followed by an oral vitamin K antagonist [3, 15, 27, 28]. Finally, while systemic and catheter-directed thrombolysis along with stent placement have been used to successfully treat catheter-associated thrombosis and thromboembolism, this evidence is limited to case reports and therefore requires further investigation [5, 6, 27].

In summary, our case of a 28-year old with IgA nephropathy who developed SVC thrombosis and subsequent SVC syndrome and pulmonary embolism highlights an under-recognized complication of HD catheter placement. When a patient with an HD catheter develops signs and symptoms related to catheter-associated thrombosis, health care providers must be mindful of other potential subsequent complications, and consider prompt workup and treatment.

## Conclusion

Superior vena cava syndrome and pulmonary embolism are potential consequences of hemodialysis catheter-related thrombosis. Practitioners should monitor for these potentially life-threatening complications in any patient with an HD catheter who develops catheter-related thrombosis. Given the increasing use of HD catheters, further investigation is needed to clarify the prevalence, pathophysiology, risk factors, prophylaxis and treatment for this potentially life-threatening complication.

## Abbreviations

HD: hemodialysis
SVC: superior vena cava
IJ: internal jugular
IV: intravenous
PE: pulmonary embolism
CVC: central venous catheter
ESRD: end-stage renal disease
CT: computed tomography
CTA: computed tomography angiography
INR: international normalized ratio
PT: prothrombin time
DVT: deep venous thrombus
DSA: digital subtraction angiography
MN: membranous nephropathy
FSGS: focal segmental glomerulosclerosis
RCT: randomized controlled trial
ACCP: American College of Chest Physicians
